# A novel method for assessing cardiac function in patients with coronary heart disease based on wrist pulse analysis

**DOI:** 10.1007/s11845-023-03341-6

**Published:** 2023-03-24

**Authors:** Wen-jie Wu, Rui Chen, Rui Guo, Jian-jun Yan, Chun-ke Zhang, Yi-qin Wang, Hai-xia Yan, Ye-qing Zhang

**Affiliations:** 1 Department of Basic Medical Science, Shanghai University of Traditional Chinese Medicine, 1200 Cailun Road, Pudong New District, Shanghai, 201203 China; 2 Institute of Intelligent Perception and Diagnosis, School of Mechanical and Power Engineering, East China University of Science and Technology, 130 Meilong Road, Xuhui District, Shanghai, 200237 China; 3 Department of Chinese Internal Medicine, Shanghai Municipal Hospital of Traditional Chinese Medicine, Shanghai University of Traditional Chinese Medicine, Shanghai, 200071 China

**Keywords:** B-type natriuretic peptide, Chronic heart failure, Coronary heart disease, Machine learning algorithm, Multiscale entropy method, Time-domain method, Wrist pulse signal

## Abstract

**Background:**

The timely assessment of B-type natriuretic peptide (BNP) marking chronic heart failure risk in patients with coronary heart disease (CHD) helps to reduce patients’ mortality.

**Objective:**

To evaluate the potential of wrist pulse signals for use in the cardiac monitoring of patients with CHD.

**Methods:**

A total of 419 patients with CHD were assigned to Group 1 (BNP < 95 pg/mL, *n* = 249), 2 (95 < BNP < 221 pg/mL, *n* = 85), and 3 (BNP > 221 pg/mL, *n* = 85) according to BNP levels. Wrist pulse signals were measured noninvasively. Both the time-domain method and multiscale entropy (MSE) method were used to extract pulse features. Decision tree (DT) and random forest (RF) algorithms were employed to construct models for classifying three groups, and the models’ performance metrics were compared.

**Results:**

The pulse features of the three groups differed significantly, suggesting different pathological states of the cardiovascular system in patients with CHD. Moreover, the RF models outperformed the DT models in performance metrics. Furthermore, the optimal RF model was that based on a dataset comprising both time-domain and MSE features, achieving accuracy, average precision, average recall, and average F1-score of 90.900%, 91.048%, 90.900%, and 90.897%, respectively.

**Conclusions:**

The wrist pulse detection technology employed in this study is useful for assessing the cardiac function of patients with CHD.

## Introduction

According to the World Health Organization, coronary heart disease (CHD) is the leading cause of death among cardiovascular diseases, and its global prevalence continues to increase [1, 2]. Chronic heart failure is a secondary complication to various cardiovascular diseases and is commonly observed in the terminal stage of CHD [3]. Accumulating epidemiological evidence has indicated that chronic heart failure leads to significantly higher clinical mortality and hospital readmission rates as a complication of CHD, and the prognosis is poor [4, 5]. Therefore, more attention must be paid to the cardiac function of patients with CHD to prevent progression to chronic heart failure.

B-type natriuretic peptide (BNP) is one of the most potent biomarkers secreted by cardiac ventricular myocytes [6, 7] and is extensively used for chronic heart failure risk stratification and adverse outcome prediction [8]. BNP levels can be used to determine the severity of myocardial ischemia and myocardial injury [9, 10]. Thus, timely BNP monitoring is for cardiac function assessment in patients with CHD to prevent progression to chronic heart failure. However, BNP is generally measured as a diagnostic and prognostic test for patients with cardiovascular diseases only during hospital admission. Therefore, wearable, noninvasive, and convenient technology for out-of-hospital monitoring of the cardiac function of patients with CHD is desirable.

Pulse diagnosis is a diagnostic skill unique to traditional Chinese medicine (TCM). By touching and sensing pulsations at the radial artery, TCM practitioners can distinguish pulse patterns and obtain pathophysiological information about the human body. However, TCM practitioners often have different interpretations of pulse patterns and diagnose conditions on the basis of their own experiences. Thus, the objectification and digitization of TCM pulse diagnosis are challenging but crucial for ensuring accuracy and repeatability. Considerable efforts have been made to realize objective pulse diagnosis through multidisciplinary based on sensing, computer science, and biomedicine. Multiple pulse detection instruments have been developed to record and analyze pulse signals at the radial artery. In addition, the processing of such signals, including preprocessing, feature extraction, and recognition, are crucial to objective pulse diagnosis. With the development of information technology and artificial intelligence (AI), techniques for extracting time-domain and nonlinear time series features from pulse signals through linear to nonlinear methods have been developed. Machine learning (ML), the core AI technology, provides intelligent solutions to medical classification problems. Wrist pulse detection technology, including signal identification and processing, has gradually been applied for classification in cardiovascular diseases [11–15]. The development of wearable healthcare devices has facilitated the application of AI to the monitoring of multiple diseases such as CVDs, corneal disease, and cancer [16–18].

In the present study, we investigated the potential of wrist pulse detection technology for monitoring the cardiac function of patients with CHD through BNP level assessment based on pulse signals.

The highlights of the present study are as follows:

1. The wrist pulse signals of patients with CHD were measured during admission, and these patients were divided into three groups based on their BNP levels at the time.
2. Pulse features were extracted through time-domain and multiscale entropy (MSE) methods and were statistically analyzed to determine the differences among the three groups.
3. Decision tree (DT) and random forest (RF) algorithms were respectively used to construct classification models based on pulse features; the performance of the different classification models was compared in terms of accuracy, precision, recall, and F1-score.
4. The RF model based on an input data set comprising both time-domain and MSE features achieved the highest performance and may be a novel means of monitoring and assessing the cardiac function of patients with CHD.

The remainder of this manuscript is arranged as follows: “Data and methods” describe the data and methods, “Results” reveal the results, “Discussion” provides the discussion, and “Conclusion” presents the conclusions.

## Data and methods

### Study patients

Patients admitted to Yueyang Hospital of Integrated Traditional Chinese and Western Medicine, Shuguang Hospital, and Shanghai Hospital of Traditional Chinese Medicine (affiliated with Shanghai University of Traditional Chinese Medicine) during 2018–2020 were enrolled in the present study if they (1) had a CHD diagnosis, (2) could cooperate with researchers to complete the symptom and sign assessments and provision of related medical history data, (3) were willing to undergo a complete clinical examination, and (4) provided written informed consent. Patients were excluded if they had (1) clinically acute myocardial infarction or acute heart failure; (2) severe obstruction with hypertrophic obstructive or dilated cardiomyopathy; (3) severe diseases such as malignant tumors or HIV; (4) severe respiratory diseases such as respiratory failure, tuberculosis, or primary bronchopulmonary carcinoma; or (5) a pacemaker.

All patients voluntarily participated in the present study and provided informed consent for us to use their personal information. All personal information was kept strictly confidential. This study was approved by the ethics committee of the Shanghai University of Traditional Chinese Medicine.

### Grouping principles

Original BNP values were converted into five level according to the expert consensus on BNP clinical application recommendations published by the American College of Cardiology [19], the patients were divided into three groups (as presented in Table 1). Patients with BNP levels < 95 pg/mL were assigned to Group 1 (*n* = 249). Patients with BNP levels > 95 pg/mL were further divided; those with BNP levels > 95 and < 221 pg/mL were assigned to Group 2 (*n* = 85), and those with BNP levels > 221 pg/mL were assigned to Group 3 (*n* = 85).

**Table 1:** BNP level groups

Levels	BNP level	Group
Level 1	<95pg/mL	Group 1
Level 2	95~221 pg/mL	Group 2
Level 3	221~459 pg/mL	Group 3
Level 4	459~1006 pg/mL	
Level 5	>1006 pg/mL	

### Measurement of pulse signals

Smart TCM-I pulse detection equipment developed by the Shanghai University of Traditional Chinese Medicine and Shanghai Asia and Pacific Computer Information System was used to collect wrist pulse signals. All patients were instructed to lie down in a quiet environment for at least 3 min and maintain a relaxed wrist. The pressure sensor of the detection equipment was attached to a wristband wrapped around the patients’ left wrist, and the pulse signals at the peak amplitude of the pulse at the left radial artery were recorded for 60 s.

### Methods of extracting features from pulse signals

The wrist pulse signals were processed for feature extraction through the time-domain and MSE methods.

#### Time-domain method

**Fig. 1:**
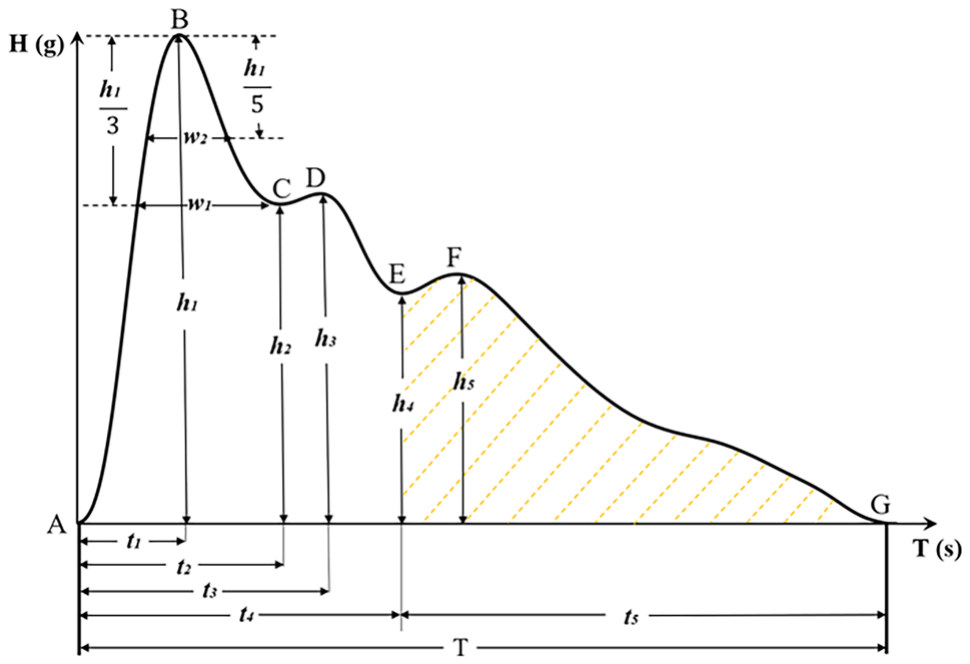
A representative pulse wave of a cardiac cycle

The time-domain method focuses on analyzing the waveform of the wrist pulse signal owing to the peak and trough points of the waveform have certain physiological significance. Seven main feature points were extracted from a representative pulse wave of a cardiac cycle shown in Fig. 1 [20], namely, the start point (A), the main wavepeak (B), the main wave gap (C), the tidal wave peak (D), the dicrotic notch (E), the dicrotic wave peak (F), and the end point (G). According to seven key feature points, three types of time-domain features, including magnitudes features, duration features, and proportional features, were finally extracted in the present study as presented in Table 2.

**Table 2:** Time-domain features of pulse signals

Feature types	Features	Feature names
Magnitude feature	*h*_1_	Amplitude of main wave peak
	*h*_2_	Amplitude of main wave gap
	*h*_3_	Amplitude of tidal wave peak
	*h*_4_	Amplitude of dicrotic notch
	*h*_5_	Amplitude of dicrotic wave
Duration feature	*t*_1_	Time from the start point to the main wave peak
	*t*_2_	Time from the start point to the main wave gap
	*t*_3_	Time from the start point to the tidal wave peak
	*t*_4_	Time from the start point to the dicrotic notch
	*t*_5_	Time from the dicrotic notch to the end point
	*T*	Pulse cycle
	*w*_1_	1/3 pulse width
	*w*_2_	1/5 pulse width
Proportional feature	*t*_1_/*T*	The ratio of the duration from the start point and the main wave peak to pusle cycle
	*t*_1_/*t*_4_	The ratio of the duration from the start point and the main wave peak to the one from the start point and the dicrotic notch
	*t*_5_/*t*_4_	The ratio of the duration from the dicrotic notch and the end point to the one from the start point and the dicrotic notch
	*w*_1_/*T*	Time ratio of 1/3 pulse width to pulse cycle
	*w*_2_/*T*	Time ratio of 1/5 pulse width to pulse cycle
	*h*_3_/*h*_1_	Amplitude ratio of tidal wave peak to main wave peak
	*h*_4_/*h*_1_	Amplitude ratio of dicrotic notch to main wave peak
	*h*_5_/*h*_1_	Amplitude ratio of dicrotic wave to main wave peak

#### MSE method

Biological systems can function on multiple time scales. Typically, biosignals are multiscaled, and their properties depend on the scale of analysis. Therefore, Costa et al. proposed the MSE method for estimating entropies from an entire set of coarse-grained time series to quantify the series’ complexity [21, 22]. The MSE method has been widely applied to quantify various biosignals, including electroencephalograpy [23], electrocardiography [24], and electromyography [25] signals. The wide application of MSE in medical diagnosis and analysis are due to its ability to correct the erratic estimations of traditional entropy-based methods [26]. Therefore, we employed the MSE method to extract MSE features of pulse signals across multiple temporal scales and quantify their complexity for different physiological and pathological states of the cardiovascular system.

MSE is an improvement of sample entropy. The procedure for the computation of MSE involves the construction of coarse-grained time series at a specific time scale and the computation of the series’ sample entropy.

**Fig. 2:**
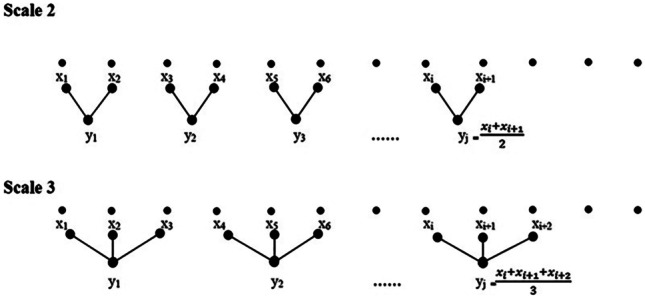
Illustration of the signal coarse-grained procedure [28]

Given a time series $x=\left\{{x}_{i}\right\},\mathrm{i}=1,\dots ,\mathrm{N}.$ The coarse-grained time series was constructed by taking the average value of consecutive data points in a non-overlapping window of data samples equal in length to time scale (Fig. 2) [27] and was described with the following formula:

1 $$\begin{array}{cc}{y}_{j}^{\left(s\right)}=\frac{1}{s}{\sum }_{i=\left(j-1\right)s+1}^{js}{x}_{i},& 1\le j\le N/s\end{array}$$

where *y*_*j*_ is a data point after a “coarse-grained” time series, *s* is the scale factor, *x*_*i*_ is a data point in the original time series, and *N* is the length of the original time series. In this paper, the maximum of scale factor *s* is 5.

In the present study, multiscale analysis was using the mean operator’s coarse-grained procedure for all pulse signals to generate the time series at a specific scale. For each scale factor *s* (*s* = 1, 2, 3, 4, 5), the sample entropy of each coarse-grained time series was calculated. MSE_*s*_ (*s* = 1, 2, 3,4,5) represents the value of sample entropy of *s*-th scale.

### ML methods

ML algorithms are the mainstream approaches to using AI for disease diagnosis and prediction. In the present study, we used two common ML algorithms—the DT and RF algorithms—to construct models for classifying patients into three groups.

**Fig. 3:**
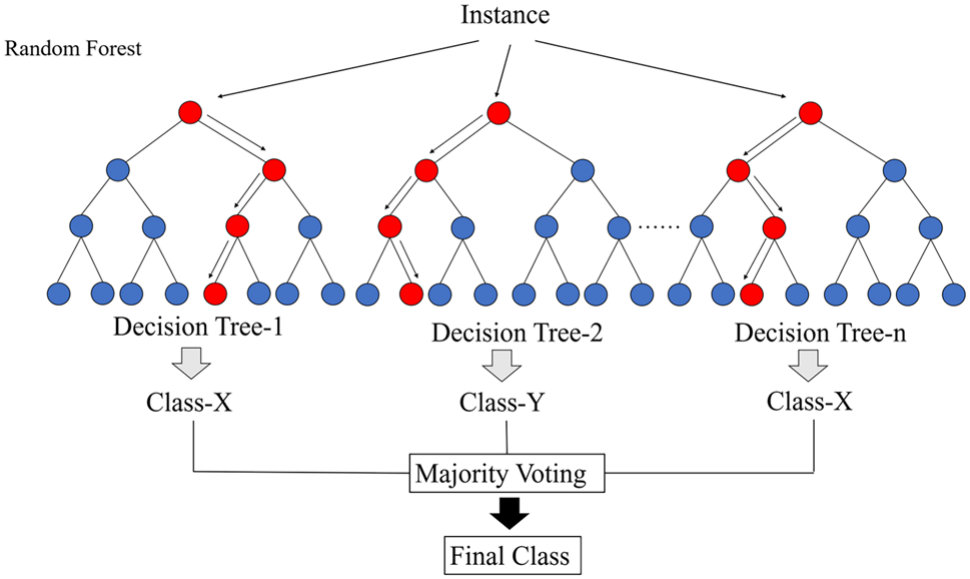
Classification process based on the RF algorithm

A DT is a tree-like regression classifier in which nodes represent attributes, links represent decision rules, and leaf nodes represent output classes. Optimal classification is achieved through the construction of a tree-like structure for feature input and creation of a unique output for every leaf [29]. However, an RF algorithm is constructed from a series of decision trees with low reciprocal correlation, and the class output by the most decision trees is the class output by the RF. The RF algorithm uses features randomly selected through bootstrap aggregation to obtain precise and stable classifications and predictions. Figure 3 illustrates how RF functions for classification [30].

### Evaluation metrics

In the present study, various classification models were constructed on the basis of three data sets and two ML algorithms. The accuracy, precision, recall, and F1-score of these methods were calculated. These were calculated as follows:

2 $$\mathrm{Accuracy}=\frac{\mathrm{number}\;\mathrm{of}\;\mathrm{all}\;\mathrm{correct}\;\mathrm{predictions}}{\mathrm{total}\;\mathrm{number}\;\mathrm{of}\;\mathrm{predictions}}$$

3 $$\mathrm{Recall}=\frac{\mathrm{number}\;\mathrm{of}\;\mathrm{correct}\;\mathrm{Group}\;X\;\mathrm{predictions}}{\mathrm{actual}\;\mathrm{number}\;\mathrm{of}\;\mathrm{patients}\;\mathrm{in}\;\mathrm{Group}\;X}$$

4 $$\mathrm{Precision}=\frac{\mathrm{number}\;\mathrm{of}\;\mathrm{correct}\;\mathrm{Group}\;\mathrm X\;\mathrm{predictions}}{\mathrm{total}\;\mathrm{number}\;\mathrm{of}\;\mathrm{Group}\;X\;\mathrm{predictions}}$$

5 $$\mathrm F1-\mathrm{score}=\frac{2\;\ast\;\mathrm{precision}\;\ast\;\mathrm{recall}}{\mathrm{precision}\;+\;\mathrm{recall}}$$

### Statistical analysis

Data analysis was performed using SPSS Statistics 25.0 (IBM, Armonk, NY, USA) to compare the pulse features and other data among the three groups.

Continuous variables were compared using the nonparametric Wilcoxon–Mann–Whitney test and are presented as median values with first and third quartiles (M (Q1, Q3)). Categorical data were compared using the chi-square test, and the results are expressed as numbers (percentages). A *P* value of < 0.05 was considered statistically significant.

## Results

### General group information

The groups’ general information (sex distributions, age, body mass index (BMI)) is presented in Table 3. The age of Group 1 was lower than those of Groups 2 and 3 (*P* < 0.05). BMI was lower in Group 3 than in Groups 1 and 2 (*P* < 0.05). The sex distributions did not differ significantly among the groups (*P* = 0.556).

**Table 3:** General information among BNP level groups

Factors	Group 1 (n = 249)	Group 2 (n = 85)	Group 3 (n = 85)	*χ*^*2*^	*P* value
Male* n* (%)	112 (45.00)	40 (47.10)	44 (51.80)	1.175	0.556
Female* n* (%)	137 (55.00)	45 (52.90)	41 (48.20)		
Age/(year)	66.73 ± 9.727	73.87 ± 8.446^*^	72.84 ± 10.053^*^	24.504	<0.001
BMI/(kg/m^2^)	24.934 ± 3.608	24.939 ± 3.960	23.737 ± 3.062^*∆^	3.795	0.023

*compared with Group 1, *P* < 0.05; ^∆^compared with Group 2, *P* < 0.05

### Comparison of time-domain features among BNP level groups

Among the time-domain features, the duration and proportional features in Table 2 can objectively reflect pathophysiological morphological changes in the pulse waveform; therefore, these features were selected for comparison among the three groups. The time-domain features that differed significantly among the three groups were *t*_5_, *T*, *h*_4_/*h*_1_, *t*_1_/*T*, *w*_1_, *w*_2_, *w*_1_/*T*, and *w*_2_/*T* (Table 4), whereas the other features were not significantly different. Here, *t*_*5*_ represents the left ventricular diastolic period; *T* represents the cardiac cycle, reflecting heart rate; *h*_*4*_*/h*_*1*_ reflects peripheral resistance; *t*_*1*_*/T* reflects the ejection function of the heart; and *w*_*1*_, *w*_*2*_, *w*_*1*_*/T*, and *w*_*2*_*/T* reflect the duration of elevated aortic pressure [31].

**Table 4:** Comparison of time-domain features among BNP level groups

Feature types	Time-domain features	Group 1 (*n* = 249)	Group 2 (*n* = 85)	Group 3 (*n* = 85)	*χ*^*2*^	*P* value
Duration feature	*t*_5_	0.413 (0.341,0.489)	0.443 (0.376, 0.509)	0.391 (0.317, 0.470)^∆∆^	9.245	0.010
	*T*	0.761 (0.680, 0.843)	0.796 (0.726, 0.857)	0.717 (0.643, 0.846)^∆∆^	9.460	0.009
Proportional feature	*h*_4_*/h*_1_	0.478 (0.412, 0.530)	0.468 (0.412, 0.518)	0.419 (0.380, 0.503)**	10.651	0.005
	*t*_1_*/T*	0.190 (0.169, 0.214)	0.183 (0.164, 0.205)	0.200 (0.175, 0.223)^∆^	7.859	0.020
	*w*_1_	0.191 (0.170, 0.213)	0.196 (0.165, 0.222)	0.183 (0.135, 0.209)*	8.600	0.014
	*w*_2_	0.143 (0.122, 0.165)	0.148 (0.115, 0.172)	0.130 (0.100, 0.161)^∆^	8.337	0.015
	*w*_1_*/T*	0.255 (0.229, 0.279)	0.250 (0.217, 0.275)	0.240 (0.209, 0.266)**	10.522	0.005
	*w*_2_*/T*	0.189 (0.167, 0.214)	0.185 (0.159, 0.213)	0.175 (0.149, 0.199)*	6.787	0.034

Data shown are M (Q1, Q3)

^*^compared with Group 1,* P* < 0.05; ^**^compared with Group 1,* P* < 0.01; ^∆^compared with Group 2, *P* < 0.05; ^∆∆^compared with Group 2,* P* < 0.01

As shown in Table 4, Group 3 had lower *h*_4_/*h*_*1*_ (*P* < 0.01), *w*_1_ (*P* < 0.05), *w*_1_/*T* (*P* < 0.01), and *w*_2_/*T* (*P* < 0.05) values than Group 1; lower* t*_5_, *T* (*P* < 0.01), and *w*_2_ (*P* < 0.05) values than Group 2; and a higher* t*_1_/*T* (*P* < 0.05) value than Group 2.

### Comparison of MSE features among BNP level groups

The MSE features of the three groups are listed in Table 5. The MSE_*s*_ (*s* = 1, 2, 3, 4, 5) values in Group 1 were higher than those in Groups 2 and 3, and the difference from Group 2 was significant (*P* < 0.05). However, the MSE_*s*_ (*s* = 1, 2, 3, 4, 5) values in Group 2 were lower than those in Group 3 (*P* < 0.05).

**Table 5:** Comparison of MSE features among BNP level groups

MSE features	Group 1 (*n* = 249)	Group 2 (*n* = 85)	Group 3 (*n* = 85)	*χ*^*2*^	*P* value
MSE_1_	0.035 (0.029, 0.039)	0.033 (0.025, 0.037)^*^	0.035 (0.028, 0.040)^∆^	8.455	0.015
MSE_2_	0.071 (0.059, 0.080)	0.067 (0.050, 0.074)^*^	0.070 (0.057, 0.083)^∆^	8.458	0.015
MSE_3_	0.108 (0.090, 0.122)	0.102 (0.076, 0.114)^*^	0.107 (0.087, 0.127)^∆^	8.458	0.015
MSE_4_	0.147 (0.122, 0.166)	0.138 (0.103, 0.155)^*^	0.146 (0.118, 0.173)^∆^	8.473	0.014
MSE_5_	0.187 (0.154, 0.212)	0.176 (0.130, 0.196)^*^	0.185 (0.149, 0.219)^∆^	8.903	0.012

Data shown are M (Q1, Q3)

^*^compared with Group 1, *P* < 0.05; ^∆^compared with Group 2,* P* < 0.05

### ML models for classifying BNP level groups

Learning the distribution pattern of an input data set aids in medical data classification using ML. In the present study, 419 samples were used to train and test the models. Because the three groups had different sample sizes, the synthetic minority oversampling technique (SMOTE) algorithm [32] was employed to equalize the sample size (*n* = 249) and avoid overfitting. The input data sets for modeling were (1) *Dataset1*, containing time-domain features and general information; (2) *Dataset2*, containing MSE features and general information; and (3) *Dataset3*, containing time-domain features, MSE features, and general information. The DT and RF algorithms were used to construct classification models based on all three datasets. The models were validated through fivefold cross-validation; that is, 80% of the pulse samples were used for training, and the remaining 20% were used for testing.

#### DT models for classifying BNP level groups

Tables 6, 7, and 8 present the classification results of DT models based on *Dataset1*, *Dataset2*, and *Dataset3*, respectively. Because the sample sizes of the three groups were balanced, accuracy and average recall were equivalent. The model based on *Dataset3* had the best performance, followed by the models based on *Dataset2* and *Dataset1*. Specifically, the model based on *Dataset3* had the highest average precision, recall, and F1-score: 78.808%, 79.116%, and 78.684%, respectively.

**Table 6:** Classification results of the DT model with *Dataset1*

Predicted classes	Actual classes (*n*)			Evaluation metrics (%)		
	Group 1	Group 2	Group 3	Precision	Recall	F1-score
Group 1	152	29	22	74.877	61.044	67.257
Group 2	49	210	13	77.206	84.337	80.614
Group 3	48	10	214	78.677	85.944	82.150
Average				76.920	77.108	76.674

**Table 7:** Classification results of the DT model with *Dataset2*

Predicted classes	Actual classes (*n*)			Evaluation metrics (%)		
	Group 1	Group 2	Group 3	Precision	Recall	F1-score
Group 1	150	22	24	76.531	60.241	67.416
Group 2	53	217	12	76.950	87.149	81.733
Group 3	46	10	213	79.182	85.542	82.239
Average				77.644	77.554	77.129

**Table 8:** Classification results for the DT model with *Dataset3*

Predicted classes	Actual classes (*n*)			Evaluation metrics (%)		
	Group 1	Group 2	Group 3	Precision	Recall	F1-score
Group 1	155	30	24	74.163	62.249	67.686
Group 2	39	215	4	83.333	86.345	84.812
Group 3	55	4	221	78.929	88.755	83.554
Average				78.808	79.116	78.684

#### RF models for classifying BNP level groups

Tables 9, 10, and 11 present the classification results of RF models based on *Dataset1*, *Dataset2*, and *Dataset3*, respectively. Because the sample sizes of the three groups were the same, accuracy and average recall were equivalent. The model based on *Dataset3* had the highest average precision, recall, and F1-score: 91.048%, 90.897%, and 90.797%, respectively.

**Table 9:** Classification results for the RF model with *Dataset1*

Predicted classes	Actual classes (*n*)			Evaluation metrics (%)		
	Group 1	Group 2	Group 3	Precision	Recall	F1-score
Group 1	194	10	11	90.233	77.912	85.521
Group 2	29	234	1	88.636	94.000	91.239
Group 3	26	5	237	88.433	95.181	91.683
Average				89.101	89.031	89.481

**Table 10:** Classification results for the RF model with *Dataset2*

Predicted classes	Actual classes (*n*)			Evaluation metrics (%)		
	Group 1	Group 2	Group 3	Precision	Recall	F1-score
Group 1	190	6	5	94.527	76.305	84.444
Group 2	34	241	3	86.691	96.787	91.461
Group 3	25	2	241	89.925	96.787	93.230
Average				90.381	89.960	89.712

**Table 11:** Classification results for the RF model with *Dataset3*

Predicted classes	Actual classes (*n*)			Evaluation metrics (%)		
	Group 1	Group 2	Group 3	Precision	Recall	F1-score
Group 1	204	7	9	92.727	81.928	86.994
Group 2	29	239	4	87.868	95.984	91.747
Group 3	16	3	236	92.549	94.779	93.651
Average				91.048	90.897	90.797

#### Performance comparison of DT and RF models

**Fig. 4:**
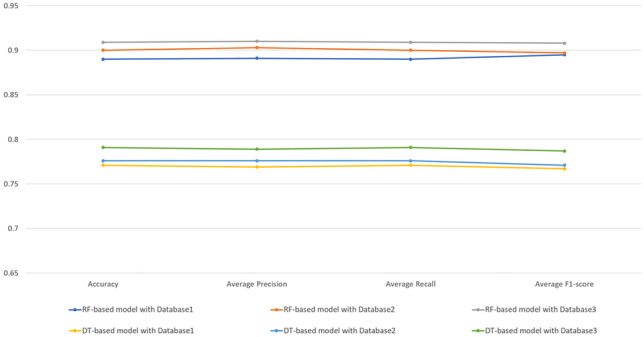
Performance comparison of DT and RF models

All RF models outperformed all DT models in terms of accuracy, average precision, average recall, and average F1-score (Fig. 4). Therefore, the RF algorithm was considered more suitable for modeling. The best performance among the RF models was achieved by that based on *Datasets3*, indicating that a combination of pulse feature types could yield more useful information than either type alone because of their reflection of different types of cardiovascular information.

## Discussion

Pulse waves are formed by the contraction of the heart and propagate outward through the arteries and blood. Various physiological factors such as blood flow, blood pressure, and the elasticity of the vascular walls of arteries and branches affect pulse waves; thus, pulse waves contain rich physiological and pathological information regarding the cardiovascular system. In TCM, pulse diagnosis is a method of measuring pulse pulsations at the radial artery to obtain human physiological and pathological information including cardiovascular information. AI facilitates medical practice and patient care and may be a powerful tool for digitalizing pulse diagnosis and improving the clinical value of the technique.

CHD complicated by chronic heart failure is common. Monitoring and identifying the emergence and development of CHD complicated by chronic heart failure and providing timely and effective clinical intervention can significantly reduce the mortality rate of patients. Current cardiac function detection methods involve coronary angiography, ultrasonic and conventional electrocardiography, and the monitoring of myocardial injury markers. These detection methods are complex and require professionals to perform, limiting their out-of-hospital application. However, wrist pulse detection can be used to conveniently, affordably, and noninvasively obtain information on the cardiovascular system through the safe monitoring of pulse signals. Therefore, we analyzed wrist pulse signals to classify patients with CHD by three BNP levels, thereby exploring the potential role of pulse signals in the real-time monitoring of cardiac function. Our findings were as follows.

The CHD patients in Group 3, who had the highest BNP levels, were older and had a lower average BMI, suggesting that cardiac function deterioration in patients with CHD is associated with old age and low BMI. A recent study observed higher mortality in lightweight patients with cardiovascular diseases than in overweight patients; this association is attributable to factors such as aging and the sarcopenia and poor nutrition of the underweight population [33].

In the present study, the time-domain features *h*_4_/*h*_1_, *w*_1_, *w*_1_/*T*, and *w*_2_/*T* were lower in Group 3 (highest BNP level) than in Group 1, possibly because patients with CHD and severe chronic heart failure have insufficient arterial blood volume. Compared with Group 2, Group 3 had lower *t*_5_ and *T* values and a higher *t*_1_/*T* value, indicating that patients with CHD and severe chronic heart failure have faster heart rates (reflected by lower *t*_5_ and *T*) and inadequate cardiac ejection capacity (reflected by higher *t*_1_/*T*). An increase in BNP indicates the exacerbation of myocardial injury, revealing poorer myocardial contractility and leading to limited cardiac ejection capacity, insufficient arterial blood volume, and lower arterial blood pressure. As myocardial systolic function declines, the heart rate may increase to compensate. These pathological changes can be reflected by changes in the time-domain features of pulse signals.

The MSE method can be used to analyze the complexity of a biological time series on different scales [34]. Larger entropy values indicate less predictability and more complex dynamics [35]. Our results showed that the MSE_*s*_ (*s* = 1, 2, 3, 4, 5) values in Groups 2 and 3 (higher BNP levels) were lower than those in Group 1 (normal BNP levels), indicating less complexity pulse signals (all *P* < 0.05). Moreover, the MSE_*s*_ values in Group 2 were significantly lower in Group 2 (moderately increased BNP levels) than in Group 3 (highest BNP levels; *P* < 0.05). This result may be due to arrhythmias in the Group 3 patients with chronic heart failure, which may lead to more random heartbeats and thus more complex pulse signals. Therefore, MSE_*s*_ values extracted from pulse signals may indicate the various pathological states associated with BNP levels.

ML, the core AI technology, is developing rapidly and provides intelligent solutions to medical classification problems. In the present study, RF and DT models based on three data sets were used to classify patients with different BNP level, and the RF models were superior to the DT models. As an ensemble learning algorithm, the RF is fast for model training, is robust to outliers, and can solve the problem of insufficient precision of a single classifier [36]. Among the RF models, the model based on the data set comprising both time-domain and MSE features achieved the best performance, indicating that a combination of pulse feature types is most informative. The time-domain features identify various morphology of pulse waveforms to obtain physiological and pathological information corresponding to different pulse morphology. MSE features represent the complexity of pulse signals because MSE is a novel nonlinear approach to measure the complexity of biological systems on multiple scales. Therefore, the two types of pulse features can complement each other to obtain rich cardiovascular information for effective classification.

## Conclusion

The time-domain and MSE features of wrist pulse signals were used to construct an RF-based model for classifying BNP level; this model achieved the superior performance with an accuracy of 90.9%, average precision of 91.048%, average recall of 90.897%, and an average F1-score of 90.797%. This study demonstrates the potential of combining wearable pulse detection technology with AI to easily assess cardiac function outside of hospitals. In the future, more types of pulse features, larger data sets, and deep learning should be considered to improve analysis.

## Data Availability

The datasets generated and analyzed during the current study are not publicly available due to the confidentiality of the data, which is an important component of the National Science Foundation of China (No. 82074332) in China, but are available from the corresponding author on reasonable request.

## Abbreviations

CHD: Coronary heart disease
BNP: B-type Natriuretic Peptide
MSE: Multiscale entropy
ML: Machine learning
DT: Decision tree
RF: Random forest
WHO: World Health Organization
TCM: Traditional Chinese medicine
SMOTE: Synthetic minority oversampling technique
BMI: Body mass index
AI: Artificial intelligence
